# Drop-out in der Alphabetisierung und Grundbildung Erwachsener – Pandemiebedingte Herausforderungen und theoretische Perspektiven

**DOI:** 10.1007/s35834-022-00338-6

**Published:** 2022-05-30

**Authors:** Marie Bickert, Jana Arbeiter, Lena Sindermann, Veronika Thalhammer

**Affiliations:** 1 Ludwig-Maximilians-Universität München, München, Deutschland; 2 Universität zu Köln, Köln, Deutschland

**Keywords:** Drop-out, Alphabetisierung, Grundbildung, Covid-19, Transformation, Soziale Ungleichheit, Drop-out, Adult basic education, Covid-19, Transformation, Social inequality

## Abstract

Das Feld der Alphabetisierung und Grundbildung Erwachsener nimmt einen zentralen Stellenwert in der Förderung gesellschaftlicher Partizipation von vulnerablen Gruppen ein. Die Covid-19 Pandemie bringt neue Transformationsprozesse mit sich, die sich auch auf die Risikofaktoren von Drop-out (Abbruch) in der Alphabetisierung und Grundbildung auswirken. Drop-out kann als Verstärker sozialer Ungleichheit beforscht werden, was insbesondere im Kontext pandemiebedingter Lernbedingungen an neuer Relevanz gewinnt. So werden in diesem Beitrag die pandemiebedingten Herausforderungen für das Feld mit besonderer Berücksichtigung von Drop-out herausgearbeitet. Anhand eines mehrstufigen Analyseverfahrens (Expert:inneninterviews & narratives Review) kann gezeigt werden, dass „Medienkompetenz und Medienzugang“, „Kontinuität und Kursstruktur“ sowie „Vertrauen und Kursbindung“ zwar als bereits bekannte Risikofaktoren für Drop-out gelten, jedoch vor dem Hintergrund pandemiebedingt veränderter Strukturen eine gestiegene Bedeutung bekommen. Pandemiebedingte Transformationsprozesse wirken wie ein Brennglas auf bereits bestehende Problemlagen im Feld der Alphabetisierung und Grundbildung. Somit kommt der pädagogischen Aufgabe der entsprechenden Gestaltung von unvorhersehbaren Veränderungen eine besondere Bedeutung zu, um der Verschärfung sozialer Ungleichheiten entgegenzuwirken.

## Abstract (en)

The field of adult basic education and literacy occupies a central position in supporting social participation of vulnerable groups. The Covid-19 pandemic brings new transformational processes that also affect the risk factors of drop-out in adult basic education and literacy. Drop-out can be studied as an amplifier of social inequality, which gains new relevance especially in the context of pandemic-related learning conditions. Thus, this paper highlights pandemic-related challenges for the field with particular attention to drop-out. Using a multi-level analysis procedure (expert interviews & narrative literature review), it can be shown that the factors “media literacy and media access”, “continuity and course structure” as well as “trust and course retention” are already known risk factors of drop-out, but take on a new significance regarding pandemic-related changes. It can be shown that pandemic-related transformational processes have an effect of a burning glass on already existing problems in the field of adult basic education and literacy. Thus, the pedagogical task of appropriately shaping unpredictable changes takes on special significance in order to counteract the increase of social inequalities.

## Einleitung

Zur Eindämmung der Covid-19 Pandemie wurden in Deutschland ab März 2020 von Bund und Ländern Leitlinien beschlossenen und kontinuierlich angepasst. Diese beinhalten u. a. Verbote oder Einschränkungen von Zusammenkünften in öffentlichen und privaten Bildungseinrichtungen des außerschulischen Bereichs. Die Durchführung von Weiterbildungen wurde während des ersten bundesweiten Lockdowns auf den virtuellen Raum beschränkt. Seitdem sind Präsenzformate durch Kontaktbeschränkungen nur begrenzt und unter Einhaltung der Auflagen möglich (u. a. Christ et al. 2021; Schmidt-Hertha 2021). Das Feld der Weiterbildung unterliegt somit neuen Transformationsprozessen, die den gesamten Weiterbildungssektor vor bislang beispiellose Herausforderungen stellen (Boeren et al. 2020). Besonders im Bereich der Alphabetisierung und Grundbildung (AuG) greifen die pandemiebedingten Veränderungen der Angebotsformate tief in die ohnehin herausfordernden Strukturen des Feldes ein. Während die Umstellung auf digitale Weiterbildungsangebote beispielsweise für viele Lehr-Lern-Settings in der Erwachsenenbildung als angemessene Anpassung an die Situation erscheint, so stellt sie für Alphabetisierungs- und Grundbildungskurse häufig eine Barriere dar, weil grundlegende Fähigkeiten im Bereich digitaler Medien für ihre Zielgruppen weniger vorausgesetzt werden können (Buddeberg und Grotlüschen 2020). Aktuelle Studien verdeutlichen, dass die zunehmende Digitalisierung auf gesamtgesellschaftlicher Ebene zur Spaltung („digital divide“ hinsichtlich Zugang, Nutzungskompetenzen und Resultate der Techniknutzung) beiträgt und die digitale Ungleichheit zwischen Gesellschaftsgruppen verstärkt (Buddeberg und Grotlüschen 2020; Kleinert et al. 2021; Rohs und Ganz 2015; White und Selwyn 2012). Die Ergebnisse einer aktuellen Umfrage machen deutlich, dass es insbesondere die gering literalisierten Erwachsenen sind, die durch eine zunehmende Digitalisierung benachteiligt werden (Institut für Demoskopie Allensbach 2021).

Durch das flächendeckende Umstrukturieren bzw. Aussetzen von Kursen steht die Weiterbildungspraxis gerade in der Alphabetisierung und Grundbildung vor der Situation, dass sich vorhandene Herausforderungen verschärfen: Nicht nur die Ansprache und Gewinnung der Teilnehmenden, sondern auch die Bemühungen zur Verstetigung der Weiterbildungsteilnahme erscheinen erschwert. Verbleib und Abbruch (Drop-out) der Teilnehmenden bekommen gleichzeitig eine neue Bedeutung. Unter Drop-out wird dabei ein Phänomen verstanden, „bei welchem Personen, die zu einer Weiterbildungsmaßnahme angemeldet sind und bis zu einem bestimmten Zeitpunkt an ihr teilnehmen, ihre Teilnahme vor regulärem Ende dieser Maßnahme einstellen“ (Hoffmann et al. 2019, S. 34). Unter der Annahme, dass eines der zentralen Ziele im Bereich der Grundbildung die Förderung gesellschaftlicher Partizipation ist (Euringer 2016), ist Drop-out nicht nur als Ausscheiden aus einem Weiterbildungsangebot zu betrachten, sondern kann sich auch als zusätzliche Barriere gesellschaftlicher Teilhabe insgesamt auswirken. Vor diesem Hintergrund kann Drop-out als Verstärker sozialer Ungleichheit beforscht werden, was insbesondere im Kontext pandemiebedingter Lernbedingungen an neuer Relevanz gewinnt. In diesem Beitrag werden die aktuellen Herausforderungen der Covid-19 Pandemie im Feld der Alphabetisierung und Grundbildung unter besonderer Berücksichtigung von Drop-out herausgearbeitet. Somit kann gezeigt werden, dass pandemiebedingte Transformationsprozesse bereits bestehende Schwierigkeiten und Problemlagen sichtbar machen und verschärfen können (Brennglas Effekt).

Unter Bezugnahme auf die theoretischen Ausführungen zur Transformationsgesellschaft von Schäffter (2001) (Kap. 2) wird zunächst die Bedeutung der Covid-19 Pandemie für die Erwachsenenbildung herausgestellt (Kap. 3). Anschließend werden aktuelle Forschungsbefunde aus dem laufenden Forschungsprojekt „Drop-out in der Alphabetisierung und Grundbildung. Analysen von Ursachen und Präventionsmöglichkeiten“ (DRAG)^1^ vorgestellt (Kap. 4). Um der Frage nachzugehen, wie pandemiebedingte Herausforderungen in den bisherigen theoretischen und empirischen Arbeiten zu Drop-out thematisiert werden, werden Befunde aus einem narrativen Review herangezogen (Abschn. 5.1). Ergänzend dazu werden aktuelle empirische Befunde zu pandemiebedingten Herausforderungen aus Sicht von Fachbereichsleitungen dargestellt (Abschn. 5.2). Die Ergebnisdarstellung dieser beiden empirischen Zugänge fokussiert drei zentrale Themenbereiche: „Medienkompetenz und Medienzugang“, „Kontinuität und Kursstruktur“ sowie „Vertrauen und Kursbindung“. Darauf wird aufbauend diskutiert, inwiefern die pandemiebedingten Herausforderungen das Drop-out Phänomen in der Alphabetisierung und Grundbildung verschärfen (Abschn. 5.3). Abschließend werden mögliche Implikationen für die Weiterbildungspraxis zum Umgang mit pandemiebedingten Drop-outs und weiterführende Forschungsperspektiven dargestellt (Kap. 6).

## Die Transformationsgesellschaft nach Schäffter (2001) als theoretische Rahmung

Die Auswirkungen der Covid-19 Pandemie legen neue Rahmenbedingungen für das Lernen Erwachsener fest. Dabei bedingen sich die strukturellen Transformationsprozesse und die Funktionsweisen der Erwachsenenbildung gegenseitig, was neue Formate für Weiterbildungsangebote erforderlich macht (Schäffter 2001). Unter zeittheoretischer Perspektive werden transformative Prozesse in der Erwachsenenbildung hinsichtlich Flexibilisierung, Beschleunigung und Entgrenzung erwachsenenpädagogischer Positionen und Praktiken diskutiert (Aldrige et al. 2020; Schmidt-Lauff 2018; Wittpoth 2020). Bei zeitdiagnostischen Ausführungen hinsichtlich des Zusammenhangs zwischen gesellschaftlichem Wandel und Erwachsenenbildung ist das Argumentationsmuster zur Transformationsgesellschaft nach Schäffter (2001) gängig (Egloff 2005). Gesellschaften unterliegen einem ständigen strukturellen Wandel, mit einer Vielzahl an konkurrierenden Antworten auf grundsätzliche Fragen der Gesellschaft. Die gesellschaftliche Transformation besteht nicht nur aus vielen einzelnen Veränderungen, sondern ist durch deren Überlagerung und gegenseitige Verstärkung gekennzeichnet, weshalb sie auch als „komplexe Gemengelage“ bezeichnet wird (Schäffter 2001, S. 45). Krisen in komplexen Systemen beschreibt Alhadeff-Jones (2021) in seinen Ausführungen zu transformativen Prozessen als unvorhersehbar. Damit werden neue Anforderungen an individuelle Orientierungsleistungen gestellt (von Felden 2014).

Der Umgang mit der Unvorhersehbarkeit einer zukünftigen Entwicklung wird in der Transformationsgesellschaft als pädagogische Aufgabe eingeordnet (Schäffter 2001). Auf einem Kontinuum zwischen Transparenz und Intransparenz der Ausgangslage und des Zielwerts unterscheidet Schäffter (2001) vier Transformationsmodelle. Zwei Modelle kennzeichnen sich durch eine zielbestimmte Transformation: Diese beiden Transformationsmodelle sind für die Herausforderungen der Covid-19 Pandemie im Feld der Alphabetisierung und Grundbildung nicht anwendbar, da die Transformationsprozesse durch zukünftige Ungewissheiten gekennzeichnet sind (Alhadeff-Jones 2021).

Anschlussfähig an pandemiebedingte Transformationsprozesse in der Alphabetisierung und Grundbildung ist die Annahme, dass der Zielwert nicht bestimmt werden kann. Schäffter (2001) stellt in diesem Zusammenhang das Selbstvergewisserungs-Modell und das Suchbewegungs-Modell auf. Beim Selbstvergewisserungs-Modell sind sowohl Ausgangs- als auch Zielwert unbekannt. Es kommt zu einer reflexiven Transformation, bei der die Dauerreflexion über Ausgangs- und Zielwert im pädagogischen Handeln institutionalisiert wird. Dagegen ist beim Suchbewegungs-Modell der Ausgangswert prinzipiell bekannt und es kommt zu einem reflexiv gesteuerten Prozessverlauf zur Bestimmung des Zielwerts. In dieser Veränderungsstruktur ist somit zwar bekannt, welche Ordnung verlassen wurde, nicht aber, wie die zukünftige Situation aussehen wird.

Diesen reflexiv gesteuerten Prozessverlauf bei der Ermittlung des unbekannten Zielwerts zeigt u. a. Rohs (2020) anhand von vier Phasen bei der Auseinandersetzung mit den Herausforderungen der Covid-19 Pandemie in der Erwachsenenbildung auf: (1) Schock-Phase, (2) Orientierung und Stabilisierung, (3) Kurzfristige Digitalisierung und (4) Systematische Flexibilisierung. Anknüpfungspunkte zur Veränderungsstruktur beim Suchbewegungs-Modell in der Alphabetisierung und Grundbildung geben Koppel und Langer (2020), die darstellen, dass den Herausforderungen der Covid-19 Pandemie nur bedingt begegnet werden kann und somit auf die Unbestimmtheit der zukünftigen Situation hinweisen. Vor dem Hintergrund erscheint das Suchbewegungs-Modell für die pandemiebedingte Festlegung neuer Rahmenbedingungen für die Erwachsenenbildung und insbesondere auch für die Alphabetisierung und Grundbildung anschlussfähig: Während die Situation vor der Krise den bekannten Ausgangswert darstellt, können zukünftige Zielwerte nur reflexiv ermittelt werden.

## Aktueller Forschungsstand: Covid-19 Pandemie und Erwachsenenbildung

Erste empirische Studien, die durch die Pandemie angestoßene und kurzfristig erkennbare Transformationsprozesse im Weiterbildungssektor erfassen, geben Hinweise für zukünftige (mittel- und langfristige) Entwicklungen und Trends. Basierend auf der Analyse von international zusammengesetzten Fokusgruppen mit Weiterbildungsexpert:innen haben Käpplinger und Lichte (2020) eine Delphi-Studie und Denninger und Käpplinger (2021) einen Literaturüberblick zu den Auswirkungen der Covid-19 Pandemie vorgelegt. Anhand der Expert:inneneinschätzung und des Literaturüberblicks lassen sich die Auswirkungen der Krise auf die Weiterbildungspraxis als (1) Disruption, (2) Beschleuniger oder (3) Brennglas beschreiben.

Empirisch lassen sich, anhand der Entwicklung der Angebote in der Weiterbildung, die durch die Covid-19 Pandemie ausgelösten *Disruptionen* bzw. Störungen in der Weiterbildungspraxis bereits gut belegen. Vor der Covid-19 Pandemie war der Präsenzunterricht in der Weiterbildung das dominierende Veranstaltungsformat (BMBF 2020). Die gängigen Präsenz-Praktiken mussten jedoch krisenbedingt vielfach unterbrochen werden (Christ et al. 2020). Onlineformate dienten während des Lockdowns der „kurzfristigen Substitution“ (BMBF 2020, S. 8) oder auch als Zusatzangebote, um die präsenzfreie Phase zu überbrücken und damit den Kontakt zu den Teilnehmer:innen zu halten (Fähser 2020). Studien belegen darüber hinaus, dass v. a. benachteiligte und gering qualifizierte Arbeitnehmer:innen während der Pandemie aufgrund der weitreichenden Schließung von Wirtschaftstätigkeiten deutlich weniger Partizipationsmöglichkeiten an non-formalen und informellen Lerngelegenheiten hatten (OECD 2021). Dies verdeutlicht den disruptiven und damit von der Weiterbildungspraxis als störend wahrgenommenen Digitalisierungs-Effekt der Covid-19 Pandemie.

Gleichzeitig wird aufgezeigt, dass die Covid-19 Pandemie in einigen Publikationen als *Beschleuniger* bestehender Prozesse des Strukturwandels in der Weiterbildung bezeichnet wird (Käpplinger und Lichte 2020; Denninger und Käpplinger 2021). V. a. zu Beginn der Krise habe die Covid-19 Pandemie „eine Entwicklung rasant beschleunigt, die bisher eher zögerlich Gestalt annahm“ (Fähser 2020, S. 43). Die Erwachsenen- und Weiterbildung befinde sich „nun in einer noch stärkeren Phase digitaler Transformation“ (Kohl und Denzl 2020, S. 25; Gnahs, 2021). Scharnberg und Krah (2020) kommen stattdessen zu einer zurückhaltenden Einschätzung: In der „transitorischen Phase“ (ebd., S. 37) habe der kurzfristige Umstieg von Bildungsangeboten auf (ausschließlich) digitale Formate in Form von „Emergency Remote Teaching“ (dt. Notfall-Fern-Lehre) weniger zu einem „Digitalisierungsschub“ (u. a. Sgodda 2021, S. 45), als zu einem „Erfahrungsschub“ (Scharnberg und Krah 2020, S. 38) geführt.

Käpplinger und Lichte (2020) und Denninger und Käpplinger (2021) zeigen drittens, dass die Covid-19 Pandemie als *Brennglas* fungiert, das bestehende Probleme eindeutiger erkennbar macht. Einerseits zeigt die Pandemie die besondere Bedeutsamkeit von Weiterbildung (insbesondere auch von Alphabetisierung und Grundbildung) hinsichtlich der Umsetzbarkeit der Pandemiebekämpfungsstrategien (Lopes und McKay 2020). Andererseits zeigte sich in vielen Ländern durch die Pandemie die Unterfinanzierung des Erwachsenenbildungssektors in Bezug auf Infrastruktur und Humanressourcen, was die Aufrechterhaltung der Qualität der Angebote, als auch die Schaffung von Strukturen zur Unterstützung der schwächsten Gruppen während der pandemiebedingten Umstellung deutlich erschwerte (ET 2020 Working Groups 2020). Die Pandemie lässt ebenso die Notwendigkeit des Ausbaus einer belastbaren technischen Infrastruktur, die prekäre Situation vieler Beschäftigter in der Erwachsenenbildung und die Bedeutung einer strategischen sowie langfristig orientierten zielgruppenspezifischen und qualitativ hochwertigen Angebotsplanung für digitale Lehre deutlicher erkennbar werden (u. a. Ehses et al. 2020; Scharnberg und Krah 2020; Schmidt-Hertha 2021). Hinsichtlich der Auswirkungen der Covid-19 Pandemie wird im Zusammenhang mit der digitalen Kluft verstärkt auf marginalisierte Personengruppen verwiesen und deren Möglichkeiten zur Abmilderung der Auswirkungen der Digitalisierung diskutiert (Sturm 2021). Anknüpfend an diese Diskussion wird v. a. bei ökonomisch Benachteiligten mit mangelnder technischer Ausstattung, bei gering Qualifizierten und bei älteren Menschen mit geringen digitalen Kompetenzen das Risiko betont, dass sich Ungleichheiten im Zugang zu Weiterbildung weiter verstärken könnten (Kohl und Denzl 2020; Schmidt-Hertha 2021).

Erste empirische Ergebnisse aus dem Bereich der Alphabetisierung und Grundbildung (Kaiper-Marquez et al. 2020; Koppel und Langer 2020) unterstreichen die besonderen pandemiebedingten Herausforderungen in diesem Weiterbildungsbereich. Koppel und Langer (2020) zeigen die spezifischen Reaktionen des Weiterbildungspersonals (reaktiv, bewahrend oder progressiv) auf die Auswirkungen der Covid-19 Pandemie auf. Dadurch lässt sich in der Alphabetisierung und Grundbildung ein bereichsspezifischer Forschungsbedarf ableiten: Aufgrund der tendenziell niedrigen Technikausstattung und der vergleichsweise besonders geringen Erfahrung mit digitalen Medien seitens der Lernenden und der Beschäftigten auf Honorarbasis scheint die Situation des Social Distancing die „‚Charakteristiken der Alphabetisierung und Grundbildung‘ stärker als zuvor bemerkbar zu machen“ (ebd., S. 35). Kaiper-Marquez et al. (2020) weisen darüber hinaus auf die zentrale Bedeutung von familiärer Grundbildung als ein pädagogisches Unterstützungssystem für geringverdienende und Migrationsfamilien während der Covid-19 Pandemie hin.

## Methodisches Vorgehen

Das Ziel des vorliegenden Beitrags bestand darin, die pandemiebedingten Herausforderungen für das Feld der Alphabetisierung und Grundbildung herauszuarbeiten, um deutlich zu machen, inwiefern ohnehin bestehende Herausforderungen durch die beschriebenen Transformationsprozesse in problematischer Weise verschärft werden können. Die ausgeführten theoretischen Überlegungen und der skizzierte Forschungsstand leiten auf zweierlei Forschungsfragen hin, die sowohl wissenschaftlich als auch im praktischen Feld von hoher Relevanz sind:

1. Welche pandemiebedingten Herausforderungen ergeben sich für die Weiterbildungspraxis in der Alphabetisierung und Grundbildung hinsichtlich des Phänomens Drop-out?
2. Wie werden die pandemiebedingten Herausforderungen in der Alphabetisierung und Grundbildung in den *bisherigen* theoretischen und empirischen Arbeiten zu Drop-out aufgegriffen?

Zur Bearbeitung der Fragestellungen wurde ein mehrstufiges Analyseverfahren gewählt, das sich auf zwei verschiedene Datenformen stützt: Interviewdaten und Publikationsdaten.

Zur Beantwortung der ersten Forschungsfrage wurden leitfadengestützte Expert:inneninterviews (Gläser und Laudel 2010) (N = 9) mit Fachbereichsleitungen von Volkshochschulen und freien Trägern zum Thema Drop-out in der Alphabetisierung und Grundbildung geführt. Das Sample wurde nach dem Prinzip der Varianzmaximierung (Patton 1990) ausgewählt (relevante Auswahlkriterien: Zentrale Akteure, öffentliche und freie Träger, Ausrichtung des Angebots hinsichtlich der Deutschsprachigkeit der Adressat:innen). Innerhalb des Samples lässt sich die Varianz hinsichtlich der Kriterien Studium/Ausbildung, Erfahrung im Berufsfeld, Aufgabenbereiche, Angebote der Institution und Erfahrung in der Funktion beschreiben. Aufgrund der pandemiebedingten Kontaktbeschränkungen zum Erhebungszeitpunkt wurden die Interviews online über Zoom geführt (Archibald et al. 2019). Die Daten wurden anhand der inhaltlich strukturierenden Inhaltsanalyse angelehnt an Kuckartz (2016) analysiert. Als Analyseeinheit für diesen Beitrag wurden Segmente durch kontextualisierte Zuordnungen und anhand der Fallzusammenfassungen zusammengestellt, in denen die Covid-19 Pandemie explizit benannt bzw. der Bezug zur Covid-19 Pandemie aus dem Kontext erschlossen wurde. Durch die regelgeleitete Systematisierung des eingegrenzten Materials wurde ein Kategoriensystem erstellt: Die Ergebnisse zu den drei zentralen Kategorien werden im Ergebnisteil der Arbeit dargestellt: Es handelt sich hierbei um die Kategorien „Medienkompetenz und Medienzugang“, „Kontinuität und Kursstruktur“ sowie „Vertrauen und Kursbindung“.

**Abb. 1:**
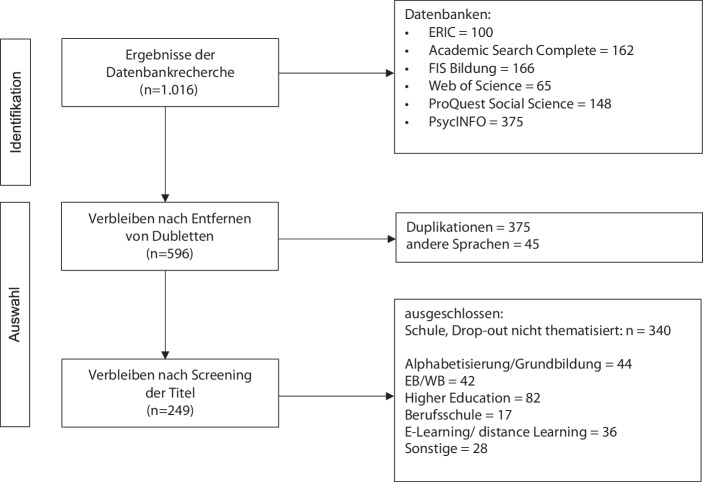
Ergebnisbaum des narrativen Reviews

Zur Beantwortung der zweiten Forschungsfrage wurden die Publikationsdaten, die in einem narrativen Review gewonnen wurden, einer strukturierenden Inhaltsanalyse (Kuckartz 2016) unterzogen. Das narrative Review (Zawacki-Richter et al. 2020) zielte insgesamt darauf ab, gängige Methoden und Theorien in der internationalen Drop-out Forschung im Feld der Alphabetisierung und Grundbildung im Kontrast zum Feld der Erwachsenen‑/Weiterbildung darzustellen. Hierfür wurden sechs einschlägige Datenbanken mit einem regelgeleiteten, intersubjektiv nachvollziehbaren Verfahren durchsucht, das der folgenden Darstellung entnommen werden kann (Abb. 1). Dazu wurden drei Suchterme zu den Oberbegriffen „Drop-out“ (Suchterm A), „Erwachsenenbildung“ (Suchterm B) und „Alphabetisierung“ (Suchterm C) in zweifacher Kombination (Suchterm A & B/Suchterm A & C) verwendet. Nach mehreren Filtervorgängen (Entfernung von: Dubletten, Treffern in anderen Sprachen als englisch und deutsch sowie Treffern mit thematischer Fehlpassung) der zunächst über 1000 Treffer wurden diese in die Bereiche Alphabetisierung/Grundbildung, Erwachsenenbildung, Higher Education, Berufsschule, E‑Learning/distance Learning sowie Sonstiges eingeteilt. Somit liegt eine strukturierte Literaturübersicht über empirische und theoretische Arbeiten für die jeweiligen Bereiche vor. Für die vorliegende Analyse wurden die Bereiche der Erwachsenenbildung, Alphabetisierung/Grundbildung, E‑Learning/distance Learning, in denen ca. 85 % der Volltexte beschafft werden konnten, einbezogen.

Aufbauend auf den Erkenntnissen aus dem narrativen Review wurde zunächst ein Leitfaden für die oben bereits genannten leitfadengestützten Expert:inneninterviews (Gläser und Laudel 2010) mit Fachbereichsleitungen entwickelt. In einem mehrstufigen und damit zirkulären Analyseverfahren wurden die aus den Expert:inneninterviews herausgearbeiteten Kernkategorien für eine erneute Analyse der für das narrative Review zusammengestellten Publikationen genutzt. Die aus dem narrativen Review vorliegenden Publikationen wurden somit für die im vorliegenden Beitrag aufgeworfenen Forschungsfragen strukturierend inhaltsanalytisch (Kuckartz 2016) analysiert. Somit wird herausgearbeitet, wie die Kategorien, die sich als pandemiebedingte Herausforderungen gezeigt haben („Medienkompetenz und Medienzugang“, „Kontinuität und Kursstruktur“ sowie „Vertrauen und Kursbindung“) in den *bisherigen* theoretischen und empirischen Arbeiten zu Drop-out bereits aufgegriffen, thematisiert und beforscht wurden: So wurde für die Zusammenführung der Ergebnisse aus den beiden empirischen Zugängen herausgearbeitet, inwiefern die pandemiebedingten Herausforderungen das Drop-out Phänomen in der Alphabetisierung und Grundbildung verschärfen (vgl. Abschn. 5.3).

## Ergebnisse

Im Folgenden werden zunächst die Ergebnisse aus dem narrativen Review vorgestellt (Abschn. 5.1) und im Anschluss daran die Befunde zu den Expert:inneninterviews (Abschn. 5.2). Die empirischen Ergebnisse werden unter Bezugnahme auf die Überlegungen zur Transformationsgesellschaft nach Schäffter (2001) hinsichtlich der Auswirkungen auf das Drop-out Phänomen in der Alphabetisierung und Grundbildung diskutiert (Abschn. 5.3).

### Befunde aus dem narrativen Review

Die folgenden Befunde aus dem narrativen Review werden entlang der Hauptkategorien „Medienkompetenz und Medienzugang“, „Kontinuität und Kursstruktur“ sowie „Vertrauen und Kursbindung“ zusammenfassend dargestellt und geben einen Überblick darüber, wie die pandemiebedingten Herausforderungen in der Alphabetisierung und Grundbildung in den bisherigen theoretischen und empirischen Arbeiten zu Drop-out thematisiert werden.

#### Medienkompetenz und Medienzugang

In den empirischen sowie theoretischen Arbeiten zu Drop-out in der Erwachsenenbildung bekommen digitale Medien je nach Forschungsfeld eine unterschiedliche Bedeutung zugesprochen. Während sich empirische Arbeiten im Bereich von E‑Learning-Angeboten verstärkt mit mangelnder digitaler Kompetenz als Ursache für Kursabbrüche befassen (Chyung et al. 1998; Utami et al. 2020), spielt dieser Faktor für Drop-out im Bereich der AuG-Forschung bislang keine große Rolle. Digitale Medien werden hier allenfalls im Rahmen der Ansprache von Adressat:innen genutzt, um Zielgruppen besser zu erreichen und Angebote zu verbreiten (Egloff et al. 2009). Als Teilnahmebarriere bzw. Einflussfaktor von Drop-out können digitale Medien im Zuge aktueller pandemiebedingter Herausforderungen (z. B. Digitalisierungsschub) relevant werden, was sich in vorliegenden Studien zum Thema noch nicht widerspiegelt.

Bremer und Pape (2019) benennen die regionale Nähe einer Weiterbildungseinrichtung als entscheidenden Faktor, der für oder gegen eine Teilnahme an einem Angebot spricht. Digitale Angebotsformate könnten dem entgegenwirken, da sie ortsunabhängig erreichbar sind. Auch (zu hohe) Kosten für die Teilnahme, die nach Schmidt (2011) eine weitere Barriere in der Erwachsenenbildung darstellen, können durch digitale Formate gesenkt werden. Voraussetzung ist hier allerdings die Verfügbarkeit und Fähigkeit zur Nutzung von Medien, da die digitalen Kenntnisse der Teilnehmenden für den Verbleib in Weiterbildungsangeboten ausschlaggebend sind (Chyung et al. 1998; Utami et al. 2020). Im Kursgeschehen der Alphabetisierung und Grundbildung spielt die soziale Interaktion für die Teilnehmendenzufriedenheit eine große Rolle (de Paepe et al. 2016), sodass diese auch im digitalen Raum entsprechend gestaltet werden sollte. Willing und Johnson (2004) finden außerdem heraus, dass die technische Unterstützung in Vorbereitung auf digitale Formate durch geschultes Personal wichtig sei. Somit kann gezeigt werden, dass medienkompetenzbezogene Gründe für Drop-out im Bildungsbereich zwar bekannt sind, diese jedoch für die Forschung in der Alphabetisierung und Grundbildung bislang unberücksichtigt sind.

#### Kontinuität und Kursstruktur

Bei der Beforschung von Drop-out in der Erwachsenenbildung werden auch die institutionellen Rahmenbedingungen von Kursen in den Blick genommen. Nach aktuellem Forschungsstand kann die Kursstruktur sowie das Format des Angebots die Entscheidung über Teilnahme, Nicht-Teilnahme oder Abbruch beeinflussen. Schmidt (2011) ermittelt neben den individuellen Bedürfnissen der Lernenden (Ebene 1) und ihren Lebenswelten (Ebene 2), weitere Ursachen für Drop-out auf institutioneller Ebene (Ebene 3). Bereits Brödel (1996) hat die Ursachen für Kursabbrüche vermehrt bei der Vorbereitung und Konzeptionierung von Angeboten verortet (vgl. auch Schäffter 1984). Daran anschließend bemerken Porras-Hernández und Salinas-Amescua in einer aktuelleren Studie von 2012, dass Angebotsstrukturen aus unterschiedlichsten Gründen häufig nicht zu den Lebensrealitäten ihrer Zielgruppen passen. Das Risiko für Drop-out verstärkt sich dabei besonders im Feld der Alphabetisierung und Grundbildung, deren Zielgruppen häufig einen prekären oder unregelmäßigen Alltag haben (Müller 2012). Thomas (1990) ermittelte in ihrer Studie Gründe für das mögliche Fernbleiben aus Grundbildungsangeboten: Die regionale Erreichbarkeit und die damit verbundene zielgruppengerechte Ansprache diese Angebote. Long und Taylor (2002) beschreiben ebenfalls den Zugang (Verfügbarkeit und Sichtbarkeit) zu Grundbildungsprogrammen als entscheidenden Faktor für Verbleib und Abbruch.

Eine aktuellere Untersuchung von Pickard (2013) bezieht sich dabei insbesondere auf die Struktur und Beschaffenheit der Kurse (Größe, Zeitraum, Typ, Intensität etc.) und merkt an, dass sich Präventionsstrategien zur Verringerung von Drop-out nur selten auf die institutionellen Rahmenbedingungen der Angebote stützen würden. Egloff et al. (2009) haben in empirischen Untersuchungen darüber hinaus das stabilisierende Moment von Alphabetisierungskursen im Alltag ihrer Teilnehmenden identifizieren können. Voraussetzung dafür sei neben der kontinuierlichen Teilnahme seitens der Lernenden auch die Beständigkeit der Angebote selbst.

#### Vertrauen und Kursbindung

Die Kategorien Vertrauen und Kursbindung werden vor allem in den empirischen Arbeiten behandelt, die individuelle, interpersonale und gruppendynamische Gründe für den Verbleib und Drop-out aufführen. Demnach sei die Zielgruppe der gering Literalisierten häufig von Stigmatisierungen und negativen Bildungserfahrungen geprägt, sodass bereits die Entscheidung für eine Weiterbildungsteilnahme und die Kursanmeldung mit Scham und Scheu verbunden seien (Steuten 2013). Patterson (2018) ordnet diese Barriere den dispositionalen Hemmnissen zu und spricht von geringem sozialem Vertrauen. In diesem Zusammenhang plädiert schon Brödel (1996) „für bildungsbiographisch reflektiertes didaktisches Handeln, um dem Drop-out begegnen zu können“ und benennt, dass bereits die sensible Ansprache im Rahmen der Veranstaltungsplanung eine große Rolle spiele (ebd. S. 25). Wenn diese Barriere überwunden sei, stelle die Teilnahme für die Zielgruppe häufig einen zentralen Stellenwert im Wochenverlauf dar, sodass die „emotionale Wertigkeit der Kursteilnahme“ (Brödel 1996, S. 27) subjektiv betrachtet sehr hoch sei. Vertrauen und Kursbindung sind also für die Teilnehmenden zentrale Einflussfaktoren für den Verbleib oder Drop-out. Auch gruppendynamische Prozesse, die die Interaktion zwischen den Teilnehmenden und den Kursleitenden und inhärente Konflikte als Gründe für den Verbleib und Drop-out kennzeichnen, werden in Betracht gezogen. Geißler (1983) weist auf die Bedeutsamkeit der Anfangssituationen von Lehr-Lernsituationen hin, die den Grundstein für das Wohlbefinden der Teilnehmenden und den Gruppenerhalt lege. Vor allem der Abgleich mit den bestehenden Erwartungshaltungen seitens der Teilnehmenden scheint hier bedeutsam (vgl. auch Darkenwald und Gavin 1987). Im Feld der Alphabetisierung und Grundbildung komme es häufig zu dem Phänomen der dauerhaften Kursbindung, sofern die sozialen und emotionalen Erwartungen an den Kurs erfüllt werden können (Egloff et al. 2009). Zudem konnten Rosenbladt und Bilger (2011) belegen, dass die enge Betreuung durch die Dozierenden einen hohen Stellenwert für die emotionale Bindung an den Kurs habe (ebd. S. 26).

### Perspektive von Expert:innen auf Drop-out und pandemiebedingte Herausforderungen

Die Darstellung der Ergebnisse aus den Expert:inneninterviews orientiert sich an den in der Analyse der Interviewdaten gebildeten Hauptkategorien „Medienkompetenz und Medienzugang“, „Kontinuität und Kursstruktur“ sowie „Vertrauen und Kursbindung“ zur Beantwortung der Forschungsfrage: „Welche pandemiebedingten Herausforderungen ergeben sich für die Weiterbildungspraxis in der Alphabetisierung und Grundbildung hinsichtlich des Phänomens Drop-out?“.

#### Medienkompetenz und Medienzugang

Das Angebot konnte von manchen Einrichtungen in Zeiten der Kontaktbeschränkungen in digitale Formate überführt werden, jedoch wurden meist Materialien per Post verschickt oder der Kontakt und die Lehre erfolgte über analoge Medien.

Eine zentrale Herausforderung beim Fernunterricht stellten potenziell fehlende Zugänge zu Medien bzw. eine als gering eingeschätzte Medienkompetenz der Teilnehmer:innen dar. Dies wird von den Interviewpartner:innen in Zusammenhang mit Abbrüchen gesetzt: „Sie haben im Prinzip ja dann das Problem, dass der Abbruch nicht mehr nur durch Motivation, sondern der kann dann auch durch externe Faktoren durch Technikbeherrschung (.) erfolgen. (.) Ja, wenn ich zwei Mal nicht in der Lage bin mich in den Kursraum einzuwählen, dann werde ich es beim dritten Mal im Zweifelsfall nicht mehr probieren“ (IP7; Abs. 56). Des Weiteren stellt die Selbststeuerung und -organisation des Lernprozesses eine Herausforderung für die Teilnehmer:innen im Fernunterricht dar. Dabei wird der Möglichkeit zur Vorbereitung des eigenständigen Lernens eine große Relevanz zugeschrieben (IP1; Abs. 67–69). Die Expert:innen verweisen hier auf die Gefahr, dass mangelnde digitale Kompetenzen neben Kompetenzen zur Lernprozesssteuerung Kursabbruchstendenzen in digitalen Angebotsformaten verstärken könnten: Bezogen auf die Drop-out Forschungsbefundlage (Chyung et al. 1998; Abschn. 5.1.1) scheint diese Befürchtung begründet. Studien zu grundlegenden Fähigkeiten im Bereich digitaler Medien der Zielgruppen in der Alphabetisierung und Grundbildung unterstreichen, dass diese i. d. R. weniger vorausgesetzt werden können (Buddeberg und Grotlüschen 2020). Vor diesem Hintergrund scheint die zurückhaltende Umstellung auf Online-Formate nachvollziehbar: Während in anderen Weiterbildungsbereichen in der Auseinandersetzung mit der Krisensituation nach der „Schock-Phase“ und der „Phase der Orientierung und Stabilisierung“ der Übergang in die dritte Phase der „kurzfristigen Digitalisierung“ (Rohs 2020) erfolgte, so wurde bei den hier befragten Expert:innen in der Alphabetisierung und Grundbildung zu dieser Phase wenig berichtet.

Neben den Kursen wurde in Formaten der Kontaktaufrechterhaltung (Eingangsberatung und Lernbegleitung) während der Covid-19 Pandemie auch nur bedingt digitale Möglichkeiten genutzt: „Pakete werden dann per Post verschickt und telefonisch wird dann noch einmal da geschaut, was für Bedarfe bestehen oder wo Unterstützung gefragt ist“ (IP2; Abs. 14). Deutlich erschweren Sprachbarrieren die Kommunikation zwischen Teilnehmer:innen und Dozent:innen bzw. Fachbereichsleitungen. Wenn in der Lernberatung aber z. B. eine Umstellung auf digitale Formate erfolgte, dann wird darin auch eine Chance zur Verstetigung gesehen und Versuche unternommen, diese neuen Formate zukünftig beizubehalten: „Das heißt, wir haben das auch auf online umgestellt und das wird in Zukunft auch hybrid […] sein“ (IP7; Abs. 16). Deutlich erkennbar ist somit zumindest in der Lernberatung die vierte Phase der „systematischen Flexibilisierung“ (Rohs 2020), in welcher versucht wird digitalisierte Angebote neben Präsenzangeboten in der Planung im Sinne einer Strategie der Flexibilisierung beizubehalten, um auf kurzfristige Entwicklungen bzgl. der Covid-19 Pandemie reagieren zu können.

#### Kontinuität und Kursstruktur

Wie bereits aufgezeigt können Ursachen für Kursabbrüche in der Vorbereitung und Konzeptionierung von Angeboten verortet werden (Brödel 1996; Schäffter 1984; Abschn. 5.1.2). Während der Covid-19 Pandemie kommt es bei den Fachbereichsleitungen zu Unsicherheiten in der Angebotsplanung und -organisation (vgl. „Schock-Phase“ nach Rohs 2020): Kurse finden „wahrscheinlich […] nicht [statt], (.) weil [es] einfach zu vage mit der Planung [ist]“ (IP5; Abs. 15). Zu den Unsicherheiten der Angebotsplanung zählt auch die Finanzierung der Kurse, die in den Einrichtungen durch Quer- oder Projektfinanzierungen unterstützt werden (IP3; Abs. 85), wodurch im Umgang mit der Krisensituation die Phase der „Orientierung und Stabilisierung“ (Rohs 2020) für die Angebote der Alphabetisierung und Grundbildung besonders erschwert schien. Außerdem kommt es zu Herausforderungen bei der Zusammenarbeit mit Netzwerkpartnern, da Partnerorganisationen ihr Angebot entweder komplett aussetzen oder sehr unterschiedliche Auflagen zur Zusammenarbeit aufstellen (IP6, Abs. 23–24).

Eine weitere Unsicherheit für die Fachbereichsleiter:innen bezieht sich auf die Lebenswelt der Teilnehmer:innen, da sich Schwangerschaft und Kinderbetreuung als Ursache von Abbrüchen pandemiebedingt verändern: Einerseits lassen sich online Formate im Vergleich zu Präsenzformaten vor dem Hintergrund von Schwangerschaft und Kinderbetreuung „leichter [umsetzen]“ (IP3; Abs. 44). Andererseits führt die pandemiebedingte Schließung von Schulen und Kinderbetreuungseinrichtungen dazu, dass Teilnehmer:innen in den Kursen „nicht mehr mit[kommen] und [deshalb abbrechen]“ (IP8; Abs. 29). Angesichts der Notwendigkeit der Einhaltung von Hygienemaßnahmen mussten Kursstrukturen neugestaltet werden. Die Gruppengrößen werden „je nach Inhalt und […] nach Räumlichkeit“ (IP1; Abs. 38) bestimmt. Die „ganz kleinen Lerngruppen von maximal vier Personen [kommen] dann im Wechsel […] in die Räumlichkeiten“ (IP6; Abs. 22). Demnach wurden nur „ein paar Leute am Tag […] in Präsenz [eingeladen]“ (IP8; Abs. 17). Somit kann gezeigt werden, dass sich durch die Covid-19 Pandemie auch Veränderungen in den sonst recht beständigen Rahmenbedingungen ergeben haben, die sich auf Angebotsplanung und Kursstrukturen beziehen.

Die durch die Covid-19 Pandemie und die damit verbundenen Hygienevorschriften ausgelösten und hier beschriebenen Unsicherheiten in der Angebotsplanung und Veränderungen in den Kursstrukturen führen teilweise zu einer Unterbrechung bzw. Einschränkung der für die Teilnehmendenbindung besonders relevanten Kontinuität. Die Fachbereichsleiter:innen beschreiben in diesem Zusammenhang die Kontinuität der Dozent:innen im Kurs, des Kursortes, der Unterrichtszeiten sowie die Kontinuität der Gruppenzusammensetzung (IP1; Abs. 46). Der besonders relevante Stabilisierungseffekt von Alphabetisierungskursen im Alltag der Teilnehmenden (Egloff et al. 2009) kann sich durch die fehlende Kontinuität in den Kursangeboten während der Covid-19 Pandemie wohl nicht entfalten. Da diese insgesamt als kritischer Einflussfaktor im Zusammenhang mit Kursabbrüchen seitens der Teilnehmer:innen gesehen wird, kann angenommen werden, dass sich dieser Effekt durch pandemiebedingte Einschränkungen verstärkt.

#### Vertrauen und Kursbindung

Die Kursanmeldungen werden von den Fachbereichsleitungen als Indikator für Kursbindung in Zeiten der Covid-19 Pandemie herangezogen. Die besondere Bedeutung der sensiblen Ansprache im Rahmen der Veranstaltungsplanung (Brödel 1996) wird somit insbesondere für die Herausforderungen der Covid-19 Pandemie deutlich: Bei der Anmeldung gibt es „sehr viele Hürden und Barrieren, weil die Anmeldung überwiegend online stattfindet und ich mich mit den Teilnehmern nicht in Person treffen kann“ (IP1; Abs. 18). Dabei wurden neue Formate für die Eingangsberatung gefunden, Neuanmeldungen und Wiederanmeldungen fanden mit Unterstützung der Dozierenden und Fachbereichsleitungen statt.

Der hohe Stellenwert der emotionalen Bindung für Verbleib und Abbruch im Bereich der Alphabetisierung und Grundbildung (Egloff et al. 2009; Rosenbladt und Bilger 2011) gewinnt auch während der Covid-19 Pandemie eine gestiegene Bedeutung. Kontakte können durch eine „enge Beziehungsarbeit“ (IP2; Abs. 38), bei der das Vertrauen zwischen Teilnehmer:innen und Dozent:innen im Mittelpunkt steht, aufrechterhalten werden. Vertrauen wurde durch „individuelle Kontakte“ (IP4; Abs. 44), Kontakte zwischen den Teilnehmer:innen und durch die Zusammenstellung von Kleingruppen hergestellt und aufrechterhalten. Dennoch haben sich teilweise Teilnehmer:innen aufgrund der Herausforderungen nicht zum Kurs (wieder)anmelden können, was dazu führt, dass „null Anmeldungen“ (IP3; Abs. 89) für Angebote vorliegen. Die Einrichtungen haben „zu vielen [Teilnehmer:innen] den Kontakt verloren“ (IP5; Abs. 56). Gründe der Teilnehmer:innen für den Kontaktabbruch ist aus Sicht der Fachbereichsleitungen die „Sorge […] sich infizieren zu können“ (IP1; Abs. 19) und die Sorge vor einem finanziellen Risiko, das mit kostenpflichtigen Angeboten einhergeht. Die Fachbereichsleitungen ordnen die Kontaktabbrüche jedoch überwiegend als „Unterbrechung oder Pausierung“ (IP1; Abs. 45) ein.

### Zusammenführung und Diskussion

Vor dem Hintergrund der theoretischen Überlegungen zur Transformationsgesellschaft nach Schäffter (2001) und unter Einbezug der empirischen Erkenntnisse lässt sich diskutieren, inwiefern die pandemiebedingten Herausforderungen das Drop-out Phänomen in der Alphabetisierung und Grundbildung verschärfen.

Im Rahmen des Suchbewegungsmodells nach Schäffter (2001) sind Transformationsprozesse als ein offener Übergang vom bekannten Ausgangswert zum unbestimmten Zielwert zu verstehen. Die in der hier vorliegenden Studie beteiligten Weiterbildungseinrichtungen reagierten auf die Herausforderungen der Covid-19 Pandemie überwiegend mit Unsicherheit bezüglich des Umgangs mit der Krisensituation v. a. im Bereich der Angebotsplanung. Das Aussetzen von Kursangeboten wirkt sich als massive Störung aus. Da weder das Ausmaß noch die Folgen der Disruption zum Zeitpunkt der Datenerhebung absehbar erscheint, war der Zielwert hier im Sinne des Suchbewegungsmodells noch völlig unbestimmt (Denninger und Käpplinger 2021; Käpplinger und Lichte 2020).

Es konnte auch gezeigt werden, dass institutionelle Barrieren**, **wie bspw. fehlende Kontinuität (Egloff et al. 2009), auch bereits vor der Covid-19 Krise als ursächlich für Drop-out ausgemacht werden konnten (Pickard 2013). Die fehlende Passung von Angebotsstrukturen zu Lebensrealitäten der Zielgruppe und Veränderungen in der Lebenswelt der Teilnehmer:innen (Porras-Hernández und Salinas-Amescua 2012) sind darüber hinaus hinsichtlich krisenbezogenen veränderten Möglichkeiten (z. B. bzgl. Kinderbetreuung) zu reflektieren. In der hier vorliegenden Studie konnten Hinweise gefunden werden, dass im Bereich „Kontinuität und Kursstruktur“ pandemiebedingte Veränderungen sich verstärkend auf bereits bekannte Drop-out-Ursachen auswirken (auch wenn eine Überprüfung der Verstärkung bzw. der Stärke der Effekte anhand des hier vorgenommenen empirischen Zugangs und auf Basis der vorliegenden Daten nicht möglich ist). Da ein zentrales Ziel von Grundbildung die Förderung gesellschaftlicher Partizipation ist und eine mögliche Verstärkung der Risikofaktoren durch die Pandemie zugleich auch eine mögliche Verschärfung sozialer Ungleichheiten mit sich bringen könnte, sind die beschriebenen Hinweise auf eine pandemiebedingte Verstärkung von potenziellen Abbruchsursachen gerade für die Zielgruppen der Alphabetisierung und Grundbildung zukünftig besonders im Blick zu behalten.

Im Suchbewegungsmodell wird der unbestimmte Zielwert innerhalb eines Möglichkeitsraums reflexiv ermittelt (Schäffter 2001). Die dargestellte Befundlage aus den Expert:inneninterviews belegt, dass die Umstellungsmöglichkeit der Angebote auf Online-Formate, die sich für andere Bereiche der Erwachsenenbildung während der Covid-19 Pandemie durchaus bewährt hat (Rohs 2020), in der Alphabetisierung und Grundbildung eher zurückhaltend erfolgte oder gar nicht möglich erschien. Als Gründe hierfür wurden die fehlenden Erfahrungswerte mit Online-Lehre, die fehlende technische Infrastruktur und die begründeten Befürchtungen bzgl. der geringen Annahmewahrscheinlichkeit seitens der überwiegend mit geringen Digitalisierungskompetenzen ausgestatten Adressat:innen angeführt. Die Ergebnisse der hier vorliegenden Studie zeigen, dass Angebote während der Covid-19 Pandemie eher unter Rückgriff auf analoge (per Telefon und Versand von Printmedien per Post) Medien durchgeführt wurden und somit den Möglichkeitsraum der Weiterbildungseinrichtungen abbilden. Eingeschränkt wird dieser Möglichkeitsraum zusätzlich durch die als gering ausgeprägt wahrgenommene Selbststeuerung und -organisation des Lernprozesses seitens der Teilnehmer:innen sowie durch Sprachbarrieren. Insbesondere die Vorbereitung der Teilnehmer:innen bezüglich der Zugangsvoraussetzungen und der Nutzung der digitalen Formate erscheint für den Verbleib der Teilnehmer:innen in den Bildungsangeboten ausschlaggebend. Zu berücksichtigen ist hier die besondere Vulnerabilität von gering literalisierten Erwachsenen hinsichtlich digitaler Grundkompetenzen: So schafft die zunehmende Digitalisierung nicht nur Partizipationshürden in verschiedenen alltäglichen Lebensbereichen (Buddeberg und Grotlüschen 2020), sondern auch in der zunehmenden digitalisierten Alphabetisierung und Grundbildung. Die dargestellten medienkompetenzbezogenen Gründe für Drop-out sind im Bildungsbereich im Allgemeinen zwar bekannt, in ihrer besonderen Brisanz im Bereich der Alphabetisierung und Grundbildung werden die Risikofaktoren „Medienkompetenz und Medienzugang“ aber erst durch die pandemiebedingten Veränderungen im Kursgeschehen sichtbar (vgl. „Brennglas“ Denninger und Käpplinger 2021; Käpplinger und Lichte 2020).

Bei der Betrachtung von Transformationsprozessen zeigt sich, dass sich einzelne Veränderungen überlagern und gegenseitig verstärken können (Schäffter 2001). Die Weiterbildungseinrichtungen reagieren auf diese komplexe Gemengelage mit einer engen Beziehungsarbeit, die durch individuelle und vertraute Kontakte zwischen Fachbereichsleiter:innen, Dozent:innen und Teilnehmer:innen umgesetzt wird. Die emotionale Kursbindung und der dahingehende Abgleich mit den bestehenden Erwartungshaltungen der Teilnehmenden ist im Bereich der Alphabetisierung und Grundbildung besonders bedeutsam und die soziale Interaktion für Verbleib und Abbruch relevant (Brödel 1996; Darkenwald und Gavin 1987; de Paepe et al. 2016). Die dargestellten Befunde zeigen die gestiegenen Bedeutung von „Vertrauen und Kursbindung“ zur Prävention von Drop-out unter den Bedingungen der Covid-19 Krise auf und sind damit an die Schlussfolgerung von Koppel und Langer (2020) anschlussfähig, dass sich die Charakteristiken der Alphabetisierung und Grundbildung durch die Covid-19 Pandemie stärker bemerkbar machen.

## Fazit und Ausblick

In dem Beitrag wurden die pandemiebedingten Herausforderungen für Drop-out in der Alphabetisierung und Grundbildung herausgearbeitet. Es konnte gezeigt werden, dass „Medienkompetenz und Medienzugang“, „Kontinuität und Kursstruktur“ sowie „Vertrauen und Kursbindung“ im Feld der Weiterbildungsforschung zwar als bereits bekannte Risikofaktoren für Drop-out gelten. Sie erhalten jedoch vor dem Hintergrund pandemiebedingter veränderter Strukturen eine gestiegene Bedeutung, die sich vor allem in deren Gestaltungsdimension zeigt: Für die Praxis der Weiterbildungseinrichtungen bedeutet dies, zwar pädagogische Transformationsprozesse aufzunehmen, jedoch entsprechende Ressourcen zu verwenden, um die Zielgruppe der gering Literalisierten nicht zurückzulassen und somit Aspekte der sozialen Ungleichheit nicht zu verschärfen. Die Covid-19 Pandemie wurde auch als Brennglas gedeutet, da sich ohnehin schon bestehende Herausforderungen der Zielgruppenansprache und -gewinnung pandemiebedingt verschärfen. Die Schlussfolgerungen sind vor dem Hintergrund vorerst zurückhaltend zu bewerten, da sie nur die Sichtweise der Fachbereichsleitungen widerspiegeln (vgl. auch Koppel und Langer 2020). In weiteren Schritten gilt es einen mehrperspektivischen Ansatz, in dem sowohl Lehrende als auch Teilnehmende einbezogen werden, zu Grunde zu legen. Des Weiteren gibt es in der Drop-out Forschung in der Erwachsenenbildung und speziell in der Alphabetisierung und Grundbildung wenig Anschluss zu staatlich angeordneten Schließungen der Weiterbildungseinrichtungen und damit verbundenen, durch die eingeschränkten Handlungsmöglichkeiten der Weiterbildungsinstitutionen ausgelösten Kursabbrüche. Auch wenn im Zusammenhang mit ökonomischen Krisen das Phänomen des „massive drop-outs“ in Schulen bereits diskutiert wurde (Jones und Hagul 2001), so steht dies für die Weiterbildungsforschung bislang noch aus.

## Footnotes

1 Das Forschungsprojekt wird mit den Förderkennzeichen W1474BFO und W147AFO vom Bundesministerium für Bildung und Forschung (BMBF) im Rahmen der Alpha-Dekade 2016–2026 gefördert.
